# Artificial intelligence in the diagnosis of obstructive sleep apnea: a scoping review

**DOI:** 10.1007/s00405-025-09377-x

**Published:** 2025-04-12

**Authors:** Miklós Kara, Zoltán Lakner, László Tamás, Viktória Molnár

**Affiliations:** 1 Department of Oto-Rhino-Laryngology and Head-Neck Surgery, Semmelweis University, Budapest, Hungary; 2 Hungarian University of Agriculture and Life Sciences, Budapest, Hungary; 3 Samarkand State Universtity, Sharof Rashidov, Univ. bld. 15, Samarkand, Usbekistan

**Keywords:** Obstructive sleep apnea, Artificial intelligence, Diagnosis, Review

## Abstract

**Purpose:**

The gold standard diagnostic modality of Obstructive Sleep Apnea (OSA) is polysomnography (PSG), which is resource-intensive, requires specialized facilities, and may not be accessible to all patients. There is a growing body of research exploring the potential of artificial intelligence (AI) to offer more accessible, efficient, and cost-effective alternatives for the diagnosis of OSA.

**Methods:**

We conducted a scoping review of studies applying AI techniques to diagnose and assess OSA in adult populations. A comprehensive search was performed in the Web of Science database using terms related to “obstructive sleep apnea,” “artificial intelligence,” “machine learning,” and related approaches.

**Results:**

A total of 344 articles met the inclusion criteria. The findings highlight various methodologies of disease evaluation, including binary classification distinguishing between OSA-positive and OSA-negative individuals in 118 articles, OSA event detection in 211 articles, severity evaluation in 38 articles, topographic diagnostic evaluation in 8 articles, and apnea-hypopnea index (AHI) estimation in 26 articles. 40 distinct types of data sources were identified. The three most prevalent data types were electrocardiography (ECG), used in 108 articles, photoplethysmography (PPG) in 62 articles, and respiratory effort and body movement in 44 articles. The AI techniques most frequently applied were convolutional neural networks (CNNs) in 104 articles, support vector machines (SVMs) in 91 articles, and K-Nearest Neighbors (KNN) in 57 articles. Of these studies, 229 used direct patient recruitment, and 115 utilized existing datasets.

**Conclusion:**

While AI demonstrates substantial potential with high accuracy rates in certain studies, challenges remain such as model transparency, validation across diverse populations, and seamless integration into clinical practice. These challenges may stem from factors such as overfitting to specific datasets, limited generalizability, and the need for standardized protocols in clinical settings.

**Supplementary Information:**

The online version contains supplementary material available at 10.1007/s00405-025-09377-x.

## Introduction

Obstructive sleep apnea (OSA) is one of the most common non-communicable diseases. It is estimated to affect 936 million people between 30 and 60 years of age worldwide [1], causing a significant reduction in quality of life due to its co-morbidities, causing premature death and numerous years of disability [2]. Its importance is rapidly increasing due to the rising prevalence of its main predisposing factor, obesity, worldwide [1]. From this follows that the diagnosis of OSA is at the forefront of research. Polysomnography is the most reliable diagnostic method [3], which requires considerable costs and logistic effort. Therefore, intensive research is being conducted to develop fast methods for screening this disease. Many centers rely on Home Sleep Apnea Testing (HSAT, typically Type III) for its reduced cost and convenience. However, HSAT cannot differentiate sleep from wake since EEG, EOG, and EMG are not monitored. This leads to the use of total recording time instead of actual sleep time to calculate the respiratory event index. Although the lack of real-time oversight in HSAT lowers costs, the absence of onsite personnel increases the risk of sensor dislodgement, resulting in poor-quality signals and additional measurement errors. Moreover, these devices cannot detect hypopneas that rely solely on cortical arousals, as recommended by the AASM Scoring Manual. Collectively, these factors often result in an underestimation of the “true” AHI, sometimes necessitating repeated studies [4]. The growing role of telemedicine highlights the importance of such methods, which can be based on the gadgets of patients, and information can be relatively easily transmitted via internet connection to centers where the analysis of these data would be the first step of screening. On the other hand, the application of modern diagnostic methods (e.g., imaging techniques) generates high-quality information that can be used for screening for OSA. In summary, there are numerous drivers for the wide-ranging application of modern statistical methods in OSA diagnosis. These methods have developed in parallel, and few studies provide a comprehensive picture of the current state of the art in this field.

This paper aims to fill this gap, provide a comprehensive overview of the current situation, and outline generalizable directions for development. We endeavored to provide a comprehensive overview, avoiding technical detail, in order to contribute to the understanding of a professional audience that is well-informed but not focused on statistical methodology. The goal was not to prepare a meta-analysis of a systematic review. Instead, the goal was to provide an overview of the diagnosis of OSA by artificial intelligence (AI) landscape. Our approach was content-based and practice-oriented. Therefore, we applied Wang’s definition of holistic intelligence: “Intelligence is the capacity of an information-processing system to adapt to its environment while operating with insufficient knowledge and resources” [5]. This definition is in line with that of High Level Expert Group on Artificial Intelligence: Artificial intelligence (AI) systems are software (and possibly also hardware) systems designed by humans that, given a complex goal, act in the physical or digital dimension by perceiving their environment through data acquisition, interpreting the collected structured or unstructured data, reasoning on the knowledge, or processing the information, derived from these data, and deciding the best action(s) to take to achieve the given goal. AI systems can either use symbolic rules or learn a numerical model, and can also adapt their behavior by analyzing how the environment is affected by their previous actions [5]. To the best of our knowledge, the current study is the first attempt to comprehensively map the landscape of AI applications in the diagnosis and assessment of OSA in adults.

## Methods

The first step of the investigation was to determine the database for our research. Recognizing that different databases had varying strengths and coverage, Web of Science database was chosen for its extensive indexing of multidisciplinary journals and its widespread use in bibliometric analyses [6]. Theoretically, the PubMed database would also have been a good choice. In this case, however, a considerable number of publications were published in journals on artificial intelligence and signal processing, outside the scope of this database.

Studies included in the review were identified through a search on the Web of Science. The exclusion of certain terms was required to increase specificity. Notably, the term **“OPTICAL”** was included in the NOT section of the search strategy. This exclusion was necessary because the acronym **“OSA”** is also commonly used for the **Optical Society of America**. The inclusion of “OSA” without exclusions led to a number of irrelevant articles on optical sciences rather than obstructive sleep apnea. The literature search was performed on October 17, 2023, using the following search query:

TS=((OSA OR (Sleep* AND Apn*)) AND (“Artificial intelligence” OR “machine learning” OR “pattern recognition” OR “neural network” OR “deep learning” OR “automatic classification” OR “expert system” OR ( “supervised” AND “learning”)) AND (“diagnos*” OR “screen*” OR “evaluat*” OR “assess*”) NOT ( child* OR pediatric* OR “Covid” OR “OPTICAL”)).

The year of publication was not a limiting factor. The earliest study identified was from 1996, and the most recent was from 2023. All non-English language articles were excluded.

The PICO framework was used to determine the eligibility criteria [7]. The PICO framework stands for Population, Intervention, Comparison, and Outcome. It helps to structure the research question and select relevant studies. This framework is based on the following research question: In adults with or at risk of OSA, how do AI techniques compare to traditional diagnostic methods in effectively diagnosing and assessing OSA?

Population (P): Adults diagnosed with or at risk of obstructive sleep apnea.

Intervention (I): Application of AI techniques—including algorithms and models that analyze complex physiological or anamnestic data to identify patterns indicative of OSA.

Comparison (C): Traditional diagnostic methods for OSA, such as polysomnography (PSG), Drug-Induced Sleep Endoscopy (DISE), or Home Sleep Apnea Test (HSAT) techniques.

**Fig. 1:**
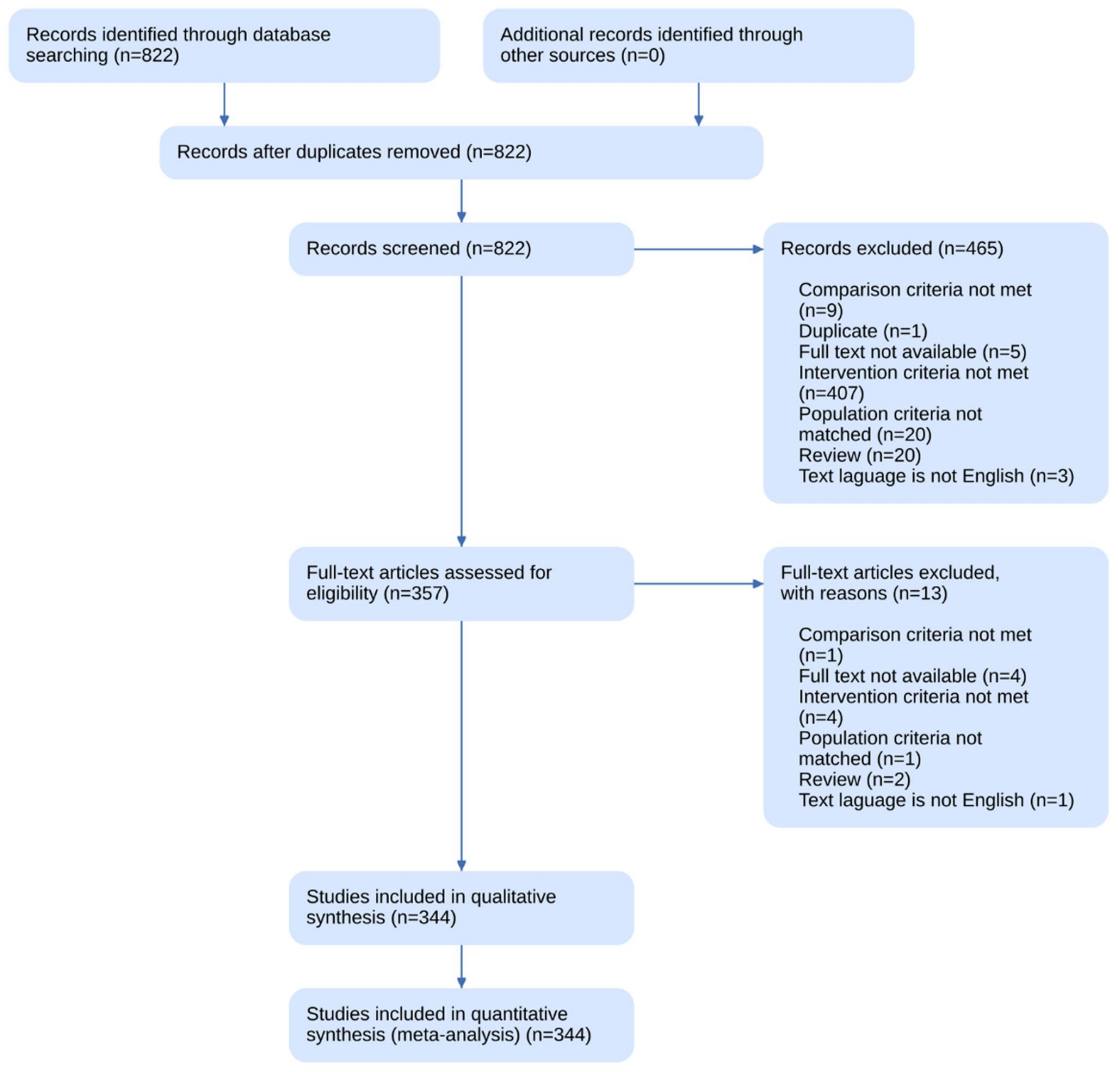
PRISMA diagram

Outcome (O): Effectiveness of diagnosing and assessing OSA, as measured by metrics such as accuracy, sensitivity, specificity, and other relevant performance indicators. Figure 1 shows the PRISMA Flow Diagram.

The review process was carried out by two independent reviewers following the PRISMA-ScR guidelines [8], with an initial calibration process involving 5% of the identified publications to ensure consistency of screening criteria. The first round thoroughly screened titles and abstracts against the eligibility criteria. The second round involved a detailed review of the full texts of the articles shortlisted in the first round. To facilitate a collaborative and reflective screening process, the review team held weekly meetings to discuss any discrepancies and refine the eligibility criteria as necessary, ensuring a comprehensive and unbiased selection of sources [9]. If consensus could not be achieved, a third reviewer was consulted to make the final decision.

The review team collaboratively developed a specific data-charting form [10]. The data chart was calibrated with 5% of all articles included. Data charting was performed independently by two research team members to minimize bias. Any discrepancies encountered during the charting process were resolved by consensus during these weekly meetings. If consensus could not be achieved, escalation to a third reviewer was used [11]. This iterative process allowed for continuous refinement of the charting form throughout the review.

## Results

All studies included used the Apnea-Hypopnea Index (AHI) as an indicator for diagnosing OSA, providing a standardized measurement for various AI applications. The review included 344 articles, 229 of which were patient-recruited studies, and 115 of which used a dataset. The size of the study population ranged from 1 to 17,748 patients, with the majority using an AHI threshold of 15/h for diagnosis. The average dataset size was approximately 679, with a median size of 70. Training and testing sets averaged 577 and 506 patients, respectively.

### Disease evaluation methods

The findings highlight a variety of methodologies for disease evaluation. Some articles employed various evaluation strategies, which means that these categories overlapped. In 118 articles, the diagnosis of OSA was treated as a binary classification problem. A classification problem is a predictive modeling exercise that aims to predict a categorical label (or class) for a given set of input features, such as a yes/no outcome [12]. In other words, classification involves assigning inputs to one of several predefined categories based on the features of the input data. The output is discrete classes: binary (yes/no) or multiclass classification. For the binary classification problem to determine whether a patient has OSA or not (OSA positive or negative), a classifier is used (increasing the number of output categories exponentially increases the number of potential classes, which requires much more computational capacities and jeopardizing the selectivity and specificity of classifications [13]). A classifier can be any AI application that categorizes input data into specific classes [14]. The diagnosis of OSA is based on AHI thresholds [15], which was > 5 AHI/h in 62 articles. A multi-class classification was present when more than one threshold was given, indicating that it was possible to assess severity. We differentiated between mild, moderate, and severe OSA with thresholds of > 5 AHI/h, > 15 AHI/h, and > 30 AHI/h, respectively. In 38 articles, AI applications were introduced to tackle this specific multi-class classification problem, giving severity evaluation as the classifier output. However, in most cases, when biometric data were used as input (e.g., ECG, PPG, or snoring sound), classifiers did not directly diagnose OSA or disease severity for the patient. In 211 articles, the classifier was used to perform per-segment classification of biometric data, and individual segments of the total recording were identified as apnea or hypopnea events. This method involves segmenting continuous biometric signals into smaller, manageable time windows (epochs) of equal length. Segment-level classifications are aggregated for the entire sleep period of the patient. This involves counting the number of segments classified as apnea or hypnoe and normalizing this count to the total sleep time to calculate AHI. The calculated AHI is used to make a clinical decision on the diagnosis and severity of OSA.

Twenty-six articles focused on AHI estimation as a direct output of the AI system. In these cases, OSA diagnosis was treated as a regression problem. A regression problem is a predictive modeling task that aims to predict a continuous numerical value based on one or more input features [12]. This approach has the significant advantage that there is no loss of information, but the practical applicability is questionable, at least in some cases.

Evaluation methods other than AHI are also emerging in the literature. Eight articles in the review attempted to use systems to determine the predominant site of collapse in the upper airway. This approach also includes a classification problem where the goal is to identify the specific location of the obstruction.

### Data collection

In the review, we identified 40 distinct data collection methods for diagnosing OSA with AI applications. These methods were grouped into 26 categories, as different data collection methods often measured the same physiological parameters. For example, respiratory effort during sleep was assessed using various techniques, such as pressure sensors, radar systems, and cameras, yet all these methods recorded the same type of data. Most of the data collected were physiological data, but in 43 cases, anamnestic data were also collected, such as body mass index (BMI), gender and age. The most prevalent sources of physiological data included electrocardiography (ECG), which was used in 108 articles, photoplethysmography (PPG) in 62 articles, and sound analysis in 29 articles. We divided the sources of physiological data into distinct categories.

### Time-series data

These data sources exist in the temporal domain. They can be utilized for time-series analysis, making them suitable for detecting patterns over time, such as apnea, hypopnea, and regular breathing events. Time-series data were the most commonly used data category encountered. This includes ECG in 108 cases where single or multiple-channel ECG was recorded at night. This high number can be traced back to The Computers in Cardiology Challenge 2000. The objective of this challenge was to advance the state of the art in apnea detection through ECG analysis. The vast majority of articles presented in the scoping review utilized the Apnea-ECG dataset from this challenge, highlighting its considerable influence on the field. This dataset is unique in that it provides minute-by-minute annotation of whether there was an apnea event, making it suitable for supervised machine learning algorithms. For instance, several studies have reported exceptionally high accuracy rates in diagnosing OSA patients using classifiers trained on this dataset, demonstrating its utility in advancing ECG-based detection methods. Kumar et al. have demonstrated that their classifier is able to diagnose OSA patients with 100% accuracy [16]. Overnight PPG recording was used in 62 articles. They achieved a high diagnostic accuracy similar to ECG analysis by extracting crucial cardio-respiratory information. Twenty-nine articles analyzed sleep breathing sounds recorded overnight. Respiratory effort and body movements were used as input in 44 instances. This was carried out by various recording devices, such as under-the-mattress radar [17], pressure plates [18], wifi antennas [19], or video cameras [20]. EEG was the source of biosignal in 26 publications. Nasal airflow was monitored in 23 cases. PSG recording was chosen as input data in 19 articles. These articles reveal the tremendous effort invested into the automatization of polysomnography. Choo et al. demonstrated a near-perfect correlation between expert scorer and the automatic system represented in their study. However, the time required by the system to analyze the overnight recording took 42.7 s compared to the 4243 s of the scoring expert [21]. Ballistocardiography (BCG) was used in five articles. BCG measures small movements of the body resulting from the contraction of the heart and the ejection of blood into the aorta, and detects these movements with sensors placed on the body or integrated into beds during sleep [22]. Bio-impedance monitoring was used in three articles. Bio-impedance of the skin surface over the chest area provided respiratory information overnight. EMG and EOG were used in two articles. Mandibular movement measuring instrumentation was used in four articles. All of these used the same SunRise system, which runs a Random Forest algorithm to predict apnea [23].

### Imaging and structural data

The following data sources provide structural or anatomical information at a single point in time, without a temporal dimension. For this reason, they cannot be used for apnea detection based on temporal patterns. Instead, they are suitable for binary OSA classification, multi-class severity evaluation problems, or regression tasks such as AHI calculation.

**Fig. 2:**
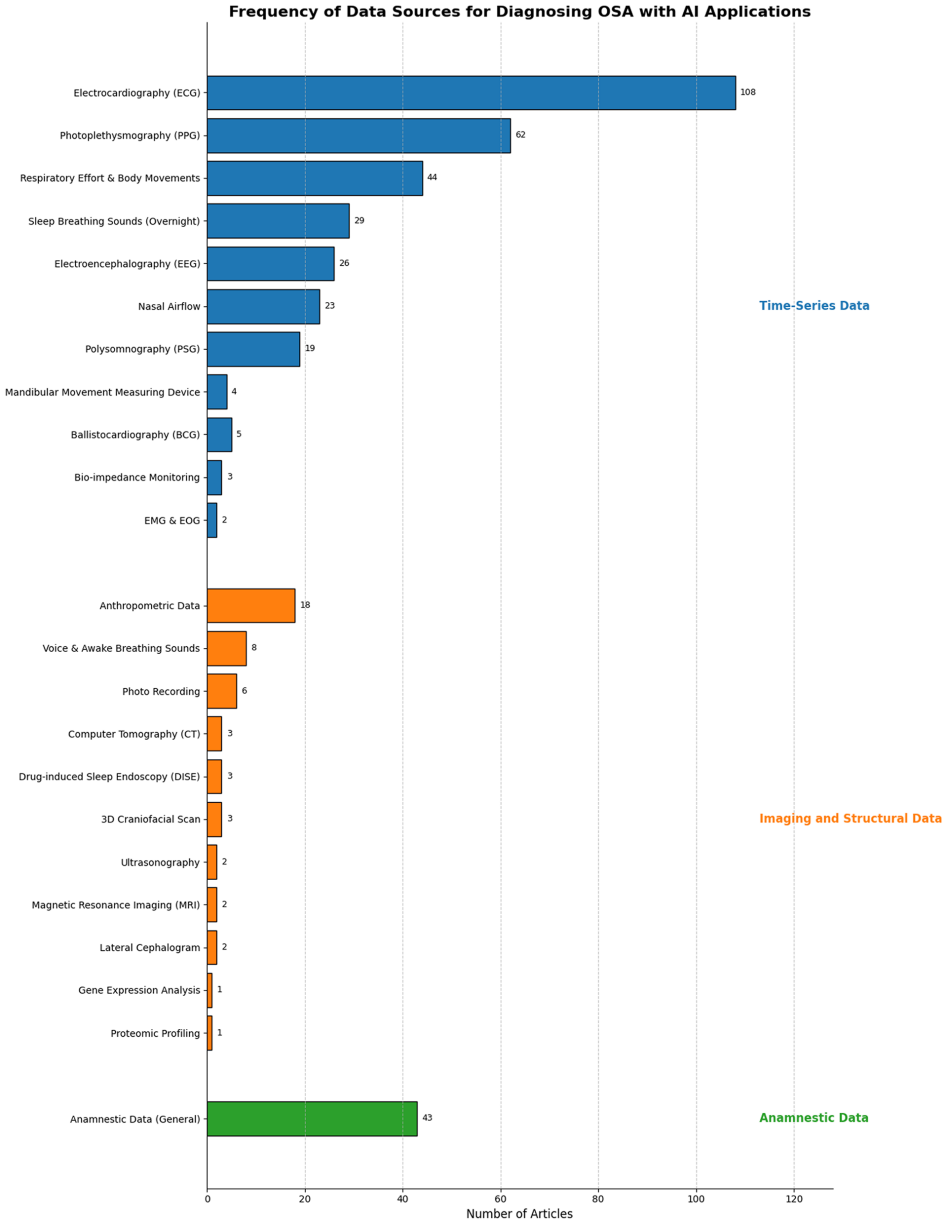
Data sources used for diagnosing OSA with AI applications

Lateral cephalograms are routinely requested by oral surgeons [24]. Two articles used lateral cephalograms. 3D Craniofacial Scan appeared in three articles. Ultrasonography was utilized in two cases. Photo recording was the choice of data collection in six instances. MRI appeared as an input variable in two studies. Computer Tomography (CT) was utilized in three articles. Drug-induced Sleep Endoscopy (DISE) was the choice of input in three instances. Anthropometric data collection was seen in 18 cases. The most common were neck and waist circumference, weight, and height. Voice and awake breathing sounds were recorded in eight cases. Gene expression analysis was applied in one article, where three gene expression datasets were used to find associations between gene expression and disease severity using feature extraction. Proteomic Profiling was used in one article. Ambati et al. found an association between 65 proteins and OSA [25]. Figure 2 shows the frequency of data sources.

### Data preprocessing and modeling workflow in AI applications for OSA diagnosis

The process of transforming raw data into meaningful insights within AI applications involves a structured, multi-stage process [26]. The initial phase involves compiling a comprehensive dataset and leveraging various data sources, such as physiological signals e.g., ECG, PPG, respiratory data, or other relevant inputs. Data pre-processing is the initial phase that involves cleaning and structuring raw data that typically contain noise and inconsistencies [12]. This pre-processing step includes eliminating noise, artifacts, and outliers, handling missing values by imputing or excluding incomplete data, normalizing signals to a consistent range, and segmenting continuous data into meaningful units (epochs), such as time windows or individual events.

Feature extraction is the next step in data transformation. In supervised learning, features are extracted manually based on their relevance to the classification or regression task. In contrast, in unsupervised learning, features help identify inherent patterns, clusters, or structures within the data and are extracted automatically [12]. This transformation from raw data to a set of measurable properties enables feeding into the classifier.

In some advanced AI applications, particularly deep learning ones, raw data can be directly fed into the model without manual feature extraction and selection [27]. This is prominently seen in convolutional neural networks (CNNs) and recurrent neural networks (RNNs), where the model architecture learns to extract and select relevant features during training.

**Fig. 3:**
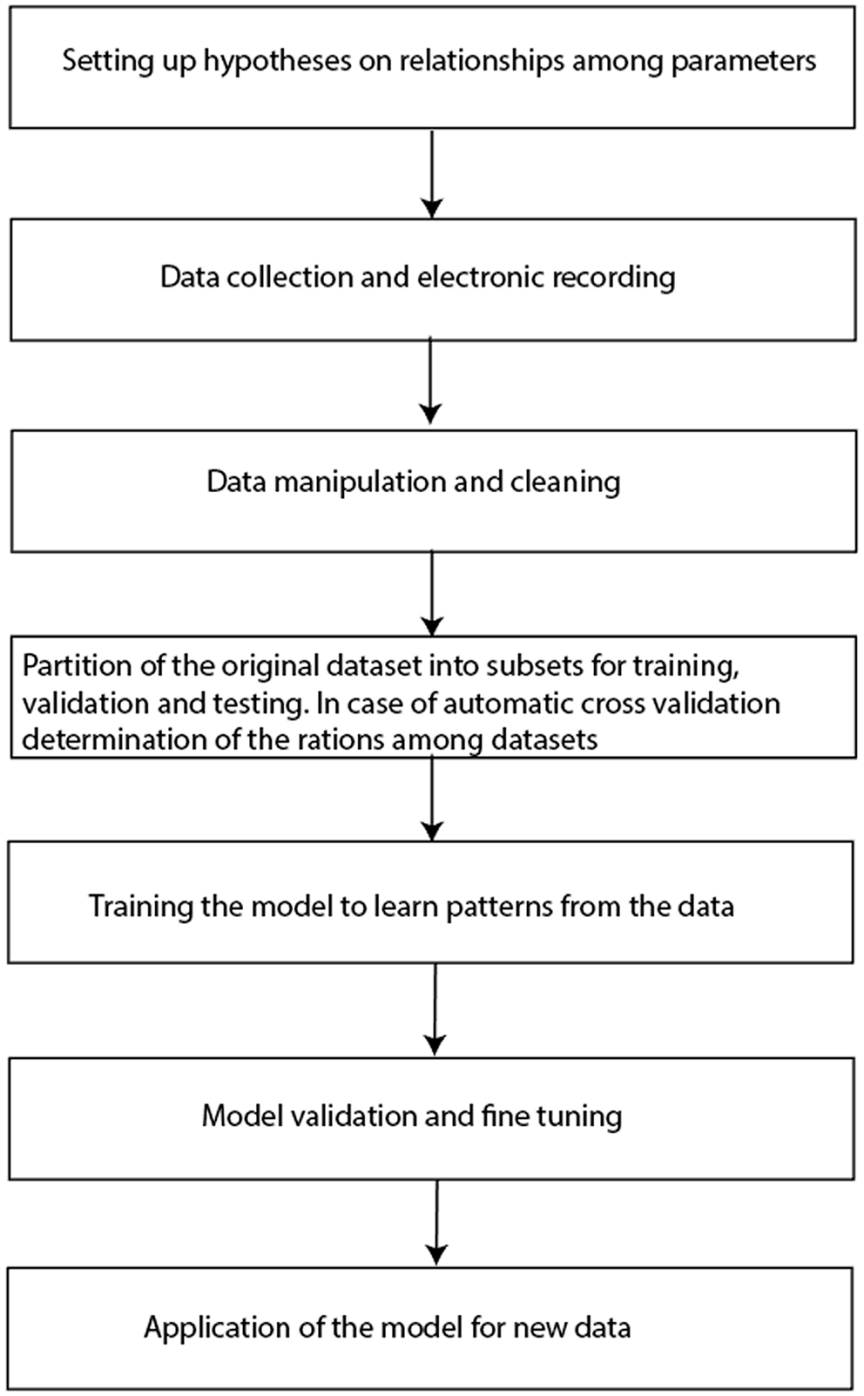
Modelling workflow

Data splitting follows feature extraction, dividing datasets into training, validation, and test sets to ensure generalizability [28]. Model training involves learning patterns from the data. This phase optimizes model parameters during supervised learning to minimize classification or regression errors. In unsupervised learning, the focus is on identifying underlying data structures, such as clusters or principal components [26]. Model validation is essential for fine-tuning the model. In supervised learning, validation sets are used to fine-tune hyperparameters and prevent overfitting [12]. In unsupervised learning, the quality of clusters, reconstruction errors, or pattern stability are assessed. The final evaluation, or model testing, provides an unbiased estimate of the performance of the model performance. Supervised learning involves testing the model on an unseen dataset to determine accuracy, precision, recall, F1-score, and other relevant metrics [29]. The modeling workflow is shown in Fig. 3.

### The AI applications

Convolutional Neural Networks (CNN) were the most frequently applied with 104 occurrences, Support Vector Machines (SVM) with 91, and K-Nearest Neighbors (KNN) with 57.

Analyzing the methods applied, it was evident that the authors used a wide range of methods, from “classical” relatively simple algorithms (e.g., logistic regression, K-Nearest Neighbors, linear discriminant analysis), which had been known and used for more than 60 years [30] to more modern methods, the results of the development of the latest decades (e.g., General Regression Neural Network [31], Random forest [32]).

**Fig. 4:**
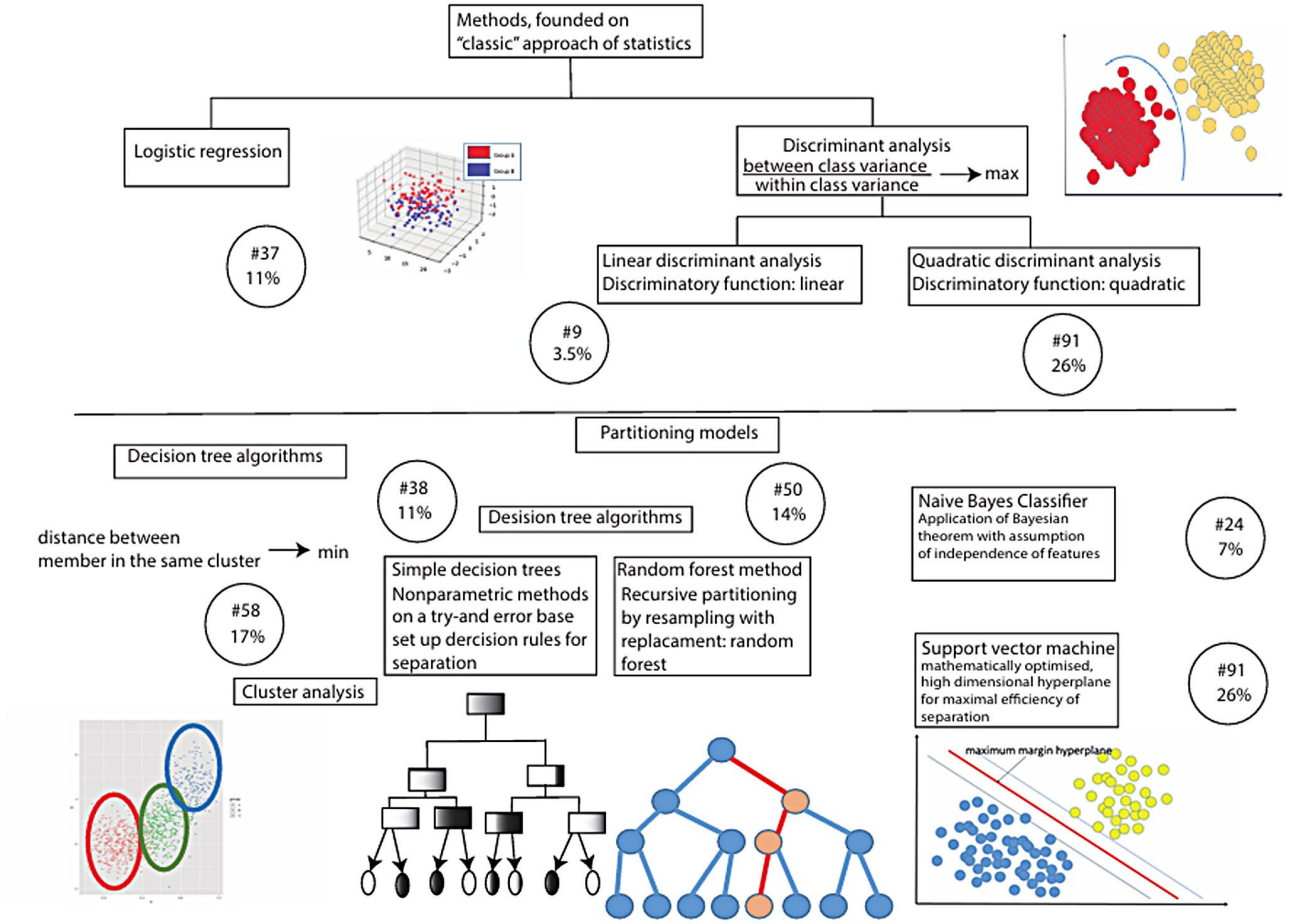
Application of various classification methods in publications I (The numbers in the circles indicate the absolute and relative frequency of the methods)

**Fig. 5:**
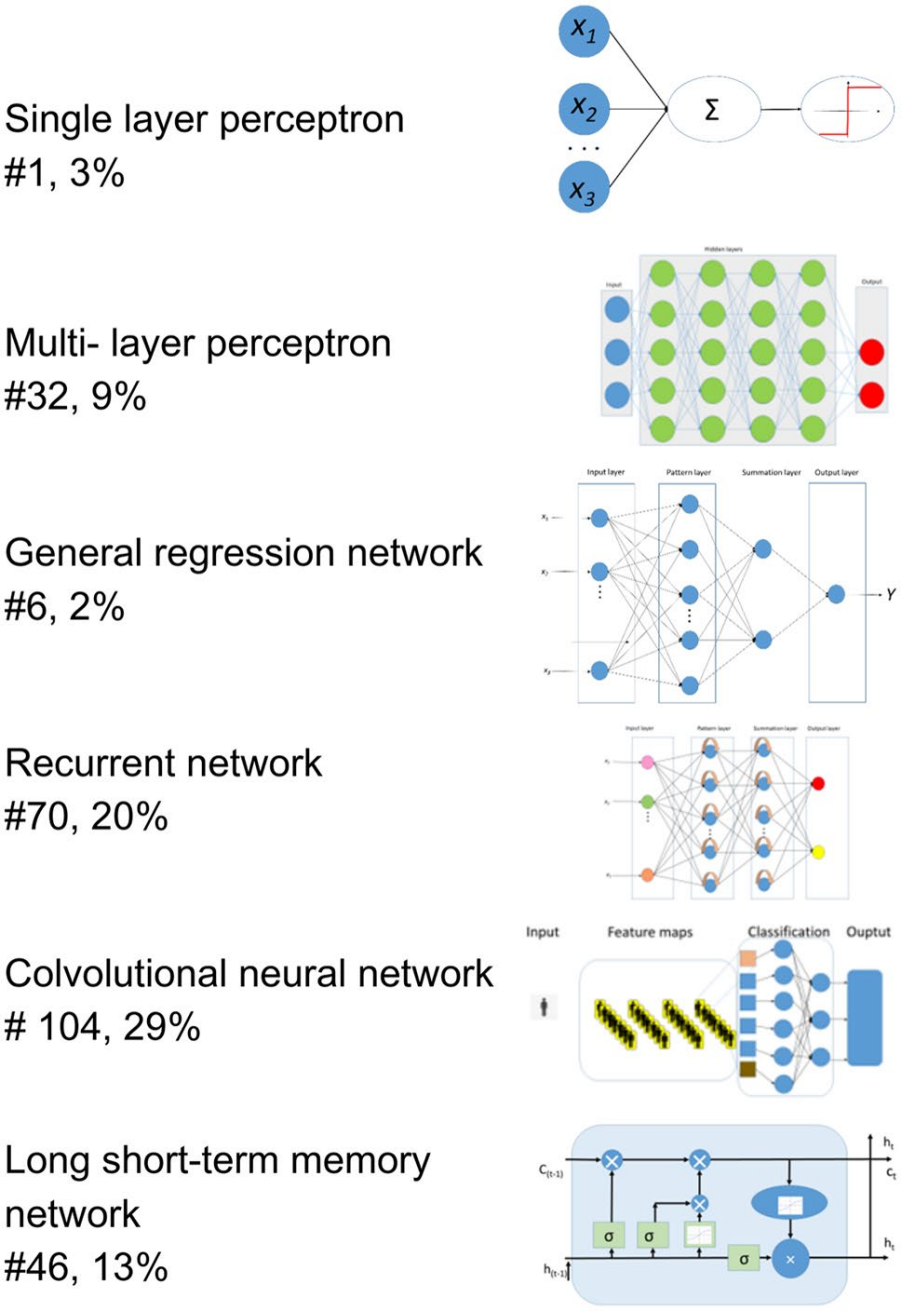
The application of neural networks in the diagnosis of OSA (The numbers in the circles indicate the absolute and relative frequency of the methods)

Earlier applications involved traditional supervised learning techniques. Supervised learning is a type of machine learning where the model is trained on a labeled dataset, which means that each training example is paired with an output label. The goal is to learn a mapping from inputs to outputs that can be used to make predictions on new, unseen data [12]. In this scoping review, we encountered Support Vector Machines (SVM) 91 times, K-Nearest Neighbors (KNN) 57 times, Logistic Regression (LR) 37 times, Random Forests (RF) 50 times, Naive Bayes (NB) 25 times, Decision Trees (DT) 37 times, Quadratic Discriminant Analysis (QDA) 9 times, and Linear Discriminant Analysis (LDA) 19 times. These algorithms were often employed as classification algorithms to categorize data into predefined classes (e.g., OSA diagnosis, severity classes, apnea/hypopnea event detection). SVMs find the optimal hyperplane that separates different classes [12]. KNN classifies data points based on the majority class among the nearest neighbors [12]. Logistic Regression provides probabilistic interpretations for binary outcomes [12]. Decision Trees provide interpretable decision rules [12]. Random Forests utilize ensemble methods to improve classification accuracy by aggregating predictions of multiple decision trees [32]. Naive Bayes applies probabilistic models to predict class membership [12]. QDA and LDA find linear combinations of features that best separate different classes [12]. Figures 4 and 5 show the machine learning techniques and neural network architectures employed in the studies included in this review and the frequency of their utilization.

As the field progressed, shallow neural networks (NN) emerged [12]. In the literature, we found one instance of a Single-Layer Perceptron (SLP) used for classification. This is the simplest form of a neural network, consisting of a single layer of output nodes connected directly to the input nodes [33]. The next step is Multi-Layer Perceptrons (MLPs), which use a hidden layer between the input and the output layers [34]. Thirty-two articles contained MLPs. These networks, with few hidden layers, are capable of capturing more complex patterns than traditional methods, while maintaining relatively low data requirements [12]. They balance model complexity and data requirements, offering improved performance over traditional algorithms while being more data-efficient than their deeper counterparts [26]. Another NN used in AHI estimation was the General Regression Neural Network (GRNN) [31]. We encountered six articles with GRNNs. Traditional neural networks, which are typically feedforward in nature, process inputs in one direction, from input to output, without cycles or feedback loops [12].

Unlike shallow neural networks, deep learning, the latest and most advanced form of neural networks, consists of multiple layers, allowing them to automatically learn and extract complex features from raw data [26, 27]. This ability to perform automatic feature extraction distinguishes deep learning from traditional shallow neural networks [12]. This makes data preparation much easier, but reduces explanatory power [26]. In the review, deep learning applications appeared in 217 instances. In particular, we saw that Convolutional Neural Networks (CNNs) were used the most often, 104 times as classifier or for feature extraction, usually both. Physiological data are often transformed into scalograms and spectrograms. CNNs are particularly effective in image processing tasks, as they automatically learn spatial hierarchies of features [26, 27]. The analysis of images derived from biosignals was a recurring theme in the literature. Recurrent Neural Networks were used in 69 instances. These networks excel in sequence prediction tasks because they can capture temporal dependencies [26]. Unlike feedforward neural networks, RNNs have connections that form cycles. This allows information to be preserved [12]. RNNs can retain information from previous inputs. This is particularly important in medical diagnostics, where understanding the temporal context of data points increases the accuracy of pattern- and anomaly detection over extended periods. In particular, Long Short-Term Memory (LSTM) networks were used in 45 articles. These networks are designed to model temporal sequences and long-range dependencies more effectively than traditional RNNs [35].

In recent years, the use of Transformers has expanded beyond natural language processing (NLP) to other areas, including the analysis of physiological data for medical diagnostics [26, 36]. The self-attention mechanism of Transformers is advantageous in handling long-term dependencies in time-series data, making them suitable for analyzing complex sequences of physiological signals such as ECG, PPG, and respiratory effort [36]. This capability is necessary for the detection of patterns indicative of conditions such as obstructive sleep apnea (OSA). The transformer is a type of model architecture first introduced by Vaswani et al. [36] for NLP tasks. However, it has since been applied in various domains thanks to its powerful sequence modeling capabilities. We found two instances in the literature where a Transformer was used to diagnose OSA.

AI systems based on different principles also appeared in the literature. We encountered Rule-based Expert Systems in three articles. These systems utilize predefined rules, typically developed by domain experts, to infer conclusions from input data [37]. Rule-based systems are particularly valuable in medical diagnostics, where expert knowledge can be codified into decision rules. These systems offer much higher explainability since the reasoning process is transparent as it is based on explicit rules [37].

The review also revealed hybrid systems that combine rule-based reasoning with machine learning techniques. These hybrid systems exploit the strengths of both approaches: the interpretability of rule-based systems and the adaptability of machine learning models [37]. For example, in some studies, rule-based systems were used to pre-process or filter data before feeding them into machine learning models or to provide an initial diagnosis that machine learning models further refine [38].

The review also highlighted the application of evolutionary algorithms and fuzzy logic systems in OSA diagnostics. Evolutionary algorithms, inspired by natural selection processes, were used in three instances to optimize feature selection and model parameters, improving the performance of machine learning models [39]. Fuzzy logic systems, which handle uncertainty and imprecision by modeling degrees of truth rather than binary true/false logic [40], appeared in six articles. These systems are useful in medical applications where data may be noisy or incomplete, allowing for more flexible and robust decision-making [37].

### Performance metrics

The evaluation of AI models in the context of OSA diagnosis involves various performance metrics. These metrics are crucial for assessing the effectiveness and reliability of the models. The most commonly reported metrics include Accuracy, Specificity (SPEC), Sensitivity (SENS), Area Under the Receiver Operating Characteristic Curve (AUC), Area Under the Precision-Recall Curve (AUPRC), F1 Score, Precision, Recall, True Positive Rate (TPR), False Positive Rate (FPR), Positive Predictive Value (PPV), Negative Predictive Value (NPV), Root Mean Square Error (RMSE), Cohen’s Kappa, Pearson’s Correlation, Spearman’s Rank Correlation, Mean Absolute Error (MAE), Mean Squared Error (MSE), Intraclass Correlation Coefficient (ICC), and generic Correlation. Detailed mathematical formulas for each of these metrics are provided in S1 Supplementary Material.

### Regression metrics

In regression problems, where the goal is to predict continuous values rather than discrete classes, the evaluation metrics differ from those used in classification tasks. Standard metrics for regression include Root Mean Square Error (RMSE), Mean Absolute Error (MAE), Mean Squared Error (MSE), Pearson’s Correlation, Spearman’s Rank Correlation, and Intraclass Correlation Coefficient (ICC).

### Comparison of classification and regression evaluations

For binary and multi-class classification problems, the focus is on correct categorization of cases into predefined classes. Metrics derived from confusion matrices are predominantly used, as the confusion matrix provides a comprehensive summary of the performance of the model, allowing for the calculation of various metrics that reflect different aspects of the predictive ability of the model, such as accuracy, precision, recall, and F1-score [41].

In contrast, regression problems are evaluated based on the accuracy and consistency of the predicted continuous values compared to the actual values. Metrics such as RMSE, MEA, and MSE directly measure the magnitude of prediction errors [12]. Correlation coefficients, such as Pearson’s and Spearman’s, assess the strength and direction of the relationship between predicted and actual values [41]. These metrics provide insights into how well the predictions of the model align with the actual values.

### Interpretation of AI performance metrics

While metrics like the F1 score often appear in AI research to demonstrate classification performance [12], their direct clinical relevance can vary. In certain screening scenarios, for instance, prioritizing sensitivity (i.e., minimizing false negatives) is paramount because missing a true OSA case can have serious health consequences. Conversely, for confirmatory purposes, specificity (i.e., minimizing false positives) may be more important to avoid unnecessary follow-up. Other metrics, such as positive or negative predictive values, also gain importance in real-world settings where disease prevalence and patient risk profiles differ from those in curated research datasets. Thus, a high F1 score alone may not capture whether the model meets the specific clinical objective—whether to rule in, rule out, or stratify disease risk [29].

Overall accuracy appeared the most frequently in the review literature. However, direct clinical utility can be limited if it does not align with real-world goals and patient populations. High accuracy alone may be misleading when the target condition has low prevalence, as a model could achieve high accuracy simply by predicting “negative” most of the time. The F1 score is the harmonic mean of precision and recall, with equal weight given to each. While this can be valuable in certain machine learning contexts, clinical scenarios often demand more targeted metrics such as PPV, NPV. Similarly, AUC can overstate performance for heavily imbalanced datasets by focusing on rank-ordering rather than absolute probability calibration [42]. In contrast, AUPRC may be more informative in settings where one class (e.g., OSA-positive) is relatively rare. In the clinical setting predictive values (PPV, NPV) are used more often, as these directly indicate how likely a positive or negative test result is to reflect true disease status [4]. Likewise, in a screening context, maximizing sensitivity to minimize missed cases could be paramount, whereas confirmatory scenarios may prioritize specificity to avoid unnecessary interventions. Some advanced applications highlight correlation coefficients or regression metrics (e.g., for AHI estimation), but these do not necessarily capture whether the predictions cross relevant clinical thresholds (e.g., AHI ≥ 15). Ultimately, when interpreting the performance metrics of AI models, readers should be critical of the results shown.

## Conclusions and policy implications

Although HSAT faces inherent limitations, AI tools offer promising solutions to enhance its diagnostic performance. Artifact detection enables machine learning models to remove poor-quality segments introduced by body movements or sensor dislodgement [43]. Data fusion from multiple nights and wearable devices alleviates the night-to-night variability issue. The optimized AI models can accurately classify apnea events from fewer channels (e.g., just respiratory effort or oximetry) [44]. By integrating these capabilities, AI-driven approaches have the potential to bring diagnostic accuracy closer to that of PSG, and ultimately expand access to OSA diagnosis, especially in settings with limited in-lab resources. Evidently, the combined application of modern patient data collection methods and their evaluation with sophisticated statistical and neural network models offers new perspectives for cost-effective preliminary screening of OSA. At the same time, it has to be recognized that there are significant overlaps and inconsistencies as a necessary consequence of the lack of coordination between different research centers, which hinders the optimal utilization of current knowledge base and methodological tools [45]. Consequently, there is a need to coordinate diverse research efforts and standardize publication and evaluation methods. The most critical steps for this type of research should be as follows:

1. Increase the proportion of publicly available primary data for further research, analysis, and testing. Modern, high-capacity data storage and retrieval systems offer a fast and easy way to exchange data [46]. Journals and other platforms for professional communication should facilitate sharing data widely, facilitating secondary data analysis using a variety of methods [45].
2. Given the substantial advancements made over the past 25 years and the increasing volume of research on OSA, it is essential to incorporate additional metrics alongside the traditional Apnea-Hypopnea Index (AHI) to assess the severity of Obstructive Sleep Apnea more accurately [47].
3. The relatively high number of studies on OSA diagnosis based on various parameters makes it necessary to systematize current knowledge and database, stimulating the preparation of meta-analysis of results [10].
4. Standardization of reporting methods on the efficiency of various classification methods could be an essential step in comparing the performance of multiple methods [48]. Existing programs, such as Stanford Medicine’s “AI in Healthcare” course and MIT’s AI and Health Care Course, are good examples of the successful integration of AI into medical education.
5. The knowledge needed to interpret the results of AI-based OSA diagnosis should be integrated into the curriculum of undergraduate and postgraduate medical education [49].

### Limitations of this review

It is important to note that this review is not a meta-analysis. Therefore, it does not include a comparative or statistical analysis of the performance metrics reported in the studies. Instead, it provides a descriptive, general overview of the various metrics used to evaluate AI models in OSA diagnosis [45]. Consequently, there is considerable potential for applying modern methods of systematic reviews and meta-analyses in future research. In addition, there is a potential for publication bias, as studies with substantial or positive findings are more likely to be published, potentially skewing the overall understanding of the topic [50]. Furthermore, the analysis was limited to articles in English, which may exclude relevant research published in other languages and thus limit the generalizability of the findings [51].

## Electronic supplementary material

Below is the link to the electronic supplementary material.

Supplementary Material 1

Supplementary Material 2

Supplementary Material 3

## References

[1] Benjafield AV et al (Aug. 2019) Estimation of the global prevalence and burden of obstructive sleep apnoea: a literature-based analysis. Lancet Respir Med 7(8):687–698. 10.1016/S2213-2600(19)30198-5 10.1016/S2213-2600(19)30198-5 PMC7007763 31300334

[2] Javaheri S, Somers VK (2011) Chapter 20 - Cardiovascular diseases and sleep apnea, in *Handbook of Clinical Neurology*, vol. 98, P. Montagna and S. Chokroverty, Eds., in Sleep Disorders Part I, vol. 98., Elsevier, pp. 327–345. 10.1016/B978-0-444-52006-7.00020-4 10.1016/B978-0-444-52006-7.00020-4 21056195

[3] Rundo JV, Downey R (2019) Polysomnography. in Handbook of clinical neurology, vol 160. Elsevier, pp 381–392. doi: 10.1016/B978-0-444-64032-1.00025-4. 10.1016/B978-0-444-64032-1.00025-4 31277862

[4] Kapur VK et al Clinical practice guideline for diagnostic testing for adult obstructive sleep apnea: an American academy of sleep medicine clinical practice guideline. J Clin Sleep Med 13(3):479–504. 10.5664/jcsm.6506 10.5664/jcsm.6506 PMC5337595 28162150

[5] AI H (2019) High-level expert group on artificial intelligence. Ethics Guidel Trust AI, 6

[6] Falagas ME, Pitsouni EI, Malietzis GA, Pappas G (2008) Comparison of pubmed, Scopus, web of science, and Google scholar: strengths and weaknesses. FASEB J 22(2):338–342. 10.1096/fj.07-9492LSF 17884971 10.1096/fj.07-9492LSF

[7] Booth A (Jan. 2006) Clear and present questions: formulating questions for evidence based practice, *Libr. Hi Tech*,24(3):355–368. 10.1108/07378830610692127

[8] PRISMA Extension for Scoping Reviews (PRISMA-ScR) Checklist and Explanation| Annals of Internal Medicine. Accessed: Sep. 21, 2024. [Online]. Available: https://www.acpjournals.org/doi/full/10.7326/M18-0850 10.7326/M18-0850 30178033

[9] Levac D, Colquhoun H, O’Brien KK (Sep. 2010) Scoping studies: advancing the methodology. *Implement. Sci.*, 5(1):69. 10.1186/1748-5908-5-69 10.1186/1748-5908-5-69 PMC2954944 20854677

[10] Arksey H, O’Malley L (Feb. 2015) Scoping studies: towards a methodological framework, *Int. J. Soc. Res. Methodol.*, 8(1):19–32. 10.1080/1364557032000119616

[11] Peters MDJ, Godfrey CM, Khalil H, McInerney P, Parker D, Soares CB Guidance for conducting systematic scoping reviews: JBI Evidence Implementation, Accessed: Sep. 21, 2024. [Online]. Available: https://journals.lww.com/ijebh/fulltext/2015/09000/Guidance_for_conducting_systematic_scoping_reviews.5.a

[12] Pattern Recognition and Machine Learning. Accessed: Sep. 21, 2024. [Online]. Available: https://link.springer.com/book/9780387310732

[13] Maglogiannis IG (2007) Emerging artificial intelligence applications in computer engineering: real word AI systems with applications in ehealth, HCI, information retrieval and pervasive technologies. IOS

[14] Murphy KP (2012) Machine learning: A probabilistic perspective. MIT Press

[15] Sateia MJ (Nov. 2014) International Classification of Sleep Disorders-Third Edition. *Chest*,146(5):1387–1394. 10.1378/chest.14-0970 10.1378/chest.14-0970 25367475

[16] Kumar CB, Mondal AK, Bhatia M, Panigrahi BK, Gandhi TK (2023) Self-Supervised representation Learning-Based OSA detection method using Single-Channel ECG signals. IEEE Trans Instrum Meas 72:1–15. 10.1109/TIM.2023.326193137323850

[17] Javaid AQ, Noble CM, Rosenberg R, Weitnauer MA (Dec. 2015) Towards Sleep Apnea Screening with an Under-the-Mattress IR-UWB Radar Using Machine Learning, in IEEE 14th International Conference on Machine Learning and Applications (ICMLA), 2015:837 -842 10.1109/ICMLA.2015.79

[18] Design and Evaluation of a Non-Contact Bed-Mounted Sensing Device for Automated In-Home Detection of Obstructive Sleep Apnea: A Pilot Study. Accessed: Sep. 21, 2024. [Online]. Available: https://www.mdpi.com/2079-6374/9/3/90

[19] Zhang Z, Conroy TB, Krieger AC, Kan EC (Apr. 2023) Detection and Prediction of Sleep Disorders by Covert Bed-Integrated RF Sensors, IEEE Trans. Biomed. Eng., 70(4):1208–1218 10.1109/TBME.2022.3212619 10.1109/TBME.2022.3212619 37815956

[20] Contactless recording of sleep apnea and periodic leg movements by nocturnal 3-D-video and subsequent visual perceptive computing| Scientific Reports. Accessed: Sep. 21, 2024. [Online]. Available: https://www.nature.com/articles/s41598-019-53050-3 10.1038/s41598-019-53050-3 PMC6856090 31727918

[21] Choo BP et al Frontiers| benchmarking performance of an automatic polysomnography scoring system in a population with suspected sleep disorders, 10.3389/fneur.2023.1123935 10.3389/fneur.2023.1123935 PMC9981786 36873452

[22] Ballistocardiography and Seismocardiography A Review of Recent Advances| IEEE Journals & Magazine| IEEE Xplore. Accessed: Sep. 21, 2024. [Online]. Available: https://ieeexplore.ieee.org/abstract/document/6916998

[23] Martinot J-B, Dong NNL, Cuthbert V, Bolly A, Gozal D, Pepin J-L (2019) Sleep apnea diagnosis supported by a neural network analysis combining single-channel mandibular movement recordings with co-morbidities and self-reported symptoms, Sep. Accessed: Sep. 21, 2024. [Online]. Available: https://erj.ersjournals.com/content/54/suppl_63/PA815

[24] Hupp JR, Tucker MR, Ellis E (2013) Contemporary oral and maxillofacial Surgery - E-Book: contemporary oral and maxillofacial Surgery - E-Book. Elsevier Health Sciences

[25] Ambati A et al (Nov. 2020) Proteomic biomarkers of sleep apnea, Sleep 43(11):zsaa086 10.1093/sleep/zsaa086 10.1093/sleep/zsaa086 PMC7686561 32369590

[26] Goodfellow I, Bengio Y, Courville A (2016) Deep learning. MIT Press

[27] LeCun Y, Bengio Y, Hinton G (2015) Deep learning. Nature 521(7553):436–444. 10.1038/nature14539 26017442

[28] Kohavi R, others (1995) A study of cross-validation and bootstrap for accuracy estimation and model selection, in *Ijcai*, Montreal, Canada, pp. 1137–1145

[29] Sokolova M, Lapalme G (2009) A systematic analysis of performance measures for classification tasks. Inf Process Manag 45(4):427–437

[30] Sebestyen GS (1962) Decision-making processes in pattern recognition (ACM monograph series). Macmillan Publishing Co., Inc.

[31] Specht DF, others (1991) A general regression neural network. IEEE Trans Neural Netw 2(6):568–576 18282872 10.1109/72.97934

[32] Breiman L (2001) Random Forests, *Mach. Learn.*, vol. 45, no. 1, Art. no. 1, Oct. 10.1023/A:1010933404324

[33] Rosenblatt F (1958) The perceptron: a probabilistic model for information storage and organization in the brain. Psychol Rev 65(6):386 13602029 10.1037/h0042519

[34] Rumelhart DE, Hinton GE, Williams RJ (1986) Learning representations by back-propagating errors, *nature*, vol. 323, no. 6088, pp. 533–536

[35] Hochreiter S (1997) Long Short-term memory. Neural Comput MIT-Press 10.1162/neco.1997.9.8.1735 9377276

[36] Vaswani A (2017) Attention is all you need. Adv Neural Inf Process Syst

[37] Russell SJ, Norvig P (2016) Artificial intelligence: a modern approach. Pearson, Accessed: Sep. 20, 2024. [Online]. Available: https://thuvienso.hoasen.edu.vn/handle/123456789/8967

[38] ElMoaqet H, Kim J, Tilbury D, Ramachandran SK, Ryalat M, Chu C-H (Nov. 2020) Gaussian mixture models for detecting sleep apnea events using single oronasal airflow record. Appl Sci 10(21): Art. 21. 10.3390/app10217889

[39] Goldberg DE (1989) E. genetic algorithms in search, optimization, and machine learning, Read. Addison-Wesley, vol. 24, pp. 27–28, 1990

[40] Zadeh LA (1965) Fuzzy sets. Inf Control 8(3):338–353

[41] Sheskin DJ (2003) Handbook of parametric and nonparametric statistical procedures: third edition, 3rd edn. Chapman and Hall/CRC, New York. 10.1201/9781420036268

[42] Davis J, Goadrich M (2006) The relationship between Precision-Recall and ROC curves, in Proceedings of the 23rd international conference on Machine learning, in ICML ’06. New York, NY, USA: Association for Computing Machinery, Jun. pp. 233–240. 10.1145/1143844.1143874

[43] Son Y et al (Aug. 2018) Automated artifact elimination of physiological signals using a deep belief network: an application for continuously measured arterial blood pressure waveforms. Inf Sci 456:145–158. 10.1016/j.ins.2018.05.018

[44] Kristiansen S, Hugaas MS, Goebel V, Plagemann T, Nikolaidis K, Liestøl K (2018) Data mining for patient friendly apnea detection. IEEE Access 6:74598–74615. 10.1109/ACCESS.2018.2882270

[45] Tenopir C et al Data Sharing by Scientists: Practices and Perceptions. 10.1371/journal.pone.0021101 10.1371/journal.pone.0021101 PMC3126798 21738610

[46] Wilkinson MD et al (Mar. 2016) The FAIR guiding principles for scientific data management and stewardship. Sci Data 3(1):160018. 10.1038/sdata.2016.18 10.1038/sdata.2016.18 PMC4792175 26978244

[47] M. A et al.(2021) Metrics of sleep apnea severity: beyond the apnea-hypopnea index, *PubMed*, Accessed: Oct. 28, 2024. [Online]. Available: https://pubmed.ncbi.nlm.nih.gov/33693939/

[48] Bossuyt PM et al (2015) STARD.,: An Updated List of Essential Items for Reporting Diagnostic Accuracy Studies. Radiology 277(3):826–832. 10.1148/radiol.2015151516 10.1148/radiol.2015151516 26509226

[49] S. A. Wartman and C. D. Combs, Medical Education Must Move From the Information Age to the… Academic Medicine, Accessed: Sep. 22, 2024. [Online]. Available: https://journals.lww.com/academicmedicine/fulltext/2018/08000/medical_education_must_move_from_the_information.15.aspx 10.1097/ACM.0000000000002044 29095704

[50] D. K, The existence of publication bias and risk factors for its occurrence, *PubMed*, (1990) Accessed: Oct. 28, 2024. [Online]. Available: https://pubmed.ncbi.nlm.nih.gov/2406472/

[51] Dh P, T A, O A, Ia A, N T (2013) Implementation research: what it is and how to do it, *PubMed*, Accessed: Oct. 28, 2024. [Online]. Available: https://pubmed.ncbi.nlm.nih.gov/24259324/

