# Supplementary material for: Artificial intelligence in the diagnosis of obstructive sleep apnea: a scoping review
Source: Eur Arch Otorhinolaryngol. 2025 Apr 12;282(10):4967–78. doi: 10.1007/s00405-025-09377-x (PMC12518446; doi:10.1007/s00405-025-09377-x)
Supplement: Supplementary file 2 — Supplementary Material 2 [file 405_2025_9377_MOESM2_ESM.pdf]

# S1 Performance Metrics

This section outlines common metrics used to evaluate the performance of AI models in diagnosing Obstructive Sleep Apnea (OSA). For an in-depth discussion of these metrics and additional evaluation measures, see [1].

## 1. Notation for Classification Metrics

- TP = True Positives
- TN = True Negatives
- FP = False Positives
- FN = False Negatives
- N = Total number of samples

**Confusion Matrix Layout:**

| | **Predicted Positive** | **Predicted Negative** |
|---|---|---|
| **Actual Positive** | TP | FN |
| **Actual Negative** | FP | TN |

## 2. Classification Metrics

### 2.1 Accuracy

$$\text{Accuracy} = \frac{\text{TP} + \text{TN}}{\text{TP} + \text{TN} + \text{FP} + \text{FN}}$$

### 2.2 Sensitivity (Recall, True Positive Rate)

$$\text{Sensitivity} = \frac{\text{TP}}{\text{TP} + \text{FN}}$$

### 2.3 Specificity (True Negative Rate)

$$\text{Specificity} = \frac{\text{TN}}{\text{TN} + \text{FP}}$$

### 2.4 Precision (Positive Predictive Value)

$$\text{Precision} = \frac{\text{TP}}{\text{TP} + \text{FP}}$$

### 2.5 Negative Predictive Value (NPV)

$$\text{NPV} = \frac{\text{TN}}{\text{TN} + \text{FN}}$$

**2.6 F1 Score**

$$\text{F1} = 2 \times \frac{\text{Precision} \times \text{Recall}}{\text{Precision} + \text{Recall}}$$

**2.7 True Positive Rate (TPR) and False Positive Rate (FPR)**

$$\text{TPR} = \frac{\text{TP}}{\text{TP} + \text{FN}} \quad , \quad \text{FPR} = \frac{\text{FP}}{\text{FP} + \text{TN}}$$

**2.8 Area Under the ROC Curve (AUC)**

$$\text{AUC} = \int_0^1 \text{TPR}(\text{FPR})\, d(\text{FPR})$$

(In practice, this is computed numerically.)

**2.9 Area Under the Precision-Recall Curve (AUPRC)**

Likewise,

$$\text{AUPRC} = \int \text{Precision}(\text{Recall})\, d(\text{Recall}),$$

which is usually approximated numerically.

**2.10 Cohen's Kappa ($\kappa$)**

$$\kappa = \frac{p_0 - p_e}{1 - p_e}$$

where

$$p_0 = \frac{\text{TP} + \text{TN}}{\text{TP} + \text{TN} + \text{FP} + \text{FN}}$$

and

$$p_e = \frac{(\text{TP} + \text{FP})(\text{TP} + \text{FN}) + (\text{FN} + \text{TN})(\text{FP} + \text{TN})}{(\text{TP} + \text{TN} + \text{FP} + \text{FN})^2}.$$

## 3. Regression Metrics

Assume a set of $N$ samples with true values $y_i$ and predicted values $\hat{y}_i$.

**3.1 Mean Squared Error (MSE)**

$$\text{MSE} = \frac{1}{N} \sum_{i=1}^{N} (y_i - \hat{y}_i)^2$$

**3.2 Root Mean Squared Error (RMSE)**

$$\text{RMSE} = \sqrt{\text{MSE}} = \sqrt{\frac{1}{N} \sum_{i=1}^{N} (y_i - \hat{y}_i)^2}$$

**3.3 Mean Absolute Error (MAE)**

$$\text{MAE} = \frac{1}{N} \sum_{i=1}^{N} |y_i - \hat{y}_i|$$

**3.4 Pearson's Correlation Coefficient (r)**

$$r = \frac{\sum_{i=1}^{N}(x_i - \bar{x})(y_i - \bar{y})}{\sqrt{\sum_{i=1}^{N}(x_i - \bar{x})^2 \ \sum_{i=1}^{N}(y_i - \bar{y})^2}}$$

**3.5 Spearman's Rank Correlation Coefficient ($\rho$)**

$$\rho = \frac{\sum_{i=1}^{N}(R(x_i) - \overline{R(x)})(R(y_i) - \overline{R(y)})}{\sqrt{\sum_{i=1}^{N}(R(x_i) - \overline{R(x)})^2 \sum_{i=1}^{N}(R(y_i) - \overline{R(y)})^2}}$$

where $R(\cdot)$ is the rank in ascending order, and $\overline{R(x)}$ is the mean rank.

**3.6 Intraclass Correlation Coefficient (ICC)** A commonly used two-way random effects model (ICC(2,1)) is defined as:

$$\text{ICC} = \frac{MS_B - MS_W}{MS_B + (k-1)\,MS_W + \frac{k}{n}(MS_R - MS_W)}$$

where

- $MS_B$ = Mean square between subjects
- $MS_W$ = Mean square within subjects
- $MS_R$ = Residual mean square
- $k$ = Number of measurements per subject
- $n$ = Number of subjects

# References


[1] Nieto, L., & Correndo, A. (2024, June 30). *Classification performance metrics and indices*. Retrieved from `https://cran.r-project.org/web/packages/metrica/vignettes/available_metrics_classification.html`
